# Radiomics approaches to predict PD-L1 and PFS in advanced non-small cell lung patients treated with immunotherapy: a multi-institutional study

**DOI:** 10.1038/s41598-023-38076-y

**Published:** 2023-07-08

**Authors:** Sevinj Yolchuyeva, Elena Giacomazzi, Marion Tonneau, Fabien Lamaze, Michele Orain, François Coulombe, Julie Malo, Wiam Belkaid, Bertrand Routy, Philippe Joubert, Venkata S. K. Manem

**Affiliations:** 1 Department of Mathematics and Computer Science, Université du Québec à Trois Rivières, Trois Rivières, Canada; 2 Institut Universitaire de Cardiologie et de Pneumologie de Québec, Quebec, Canada; 3 Centre de Recherche du Centre Hospitalier Universitaire de Montréal, Montréal, Canada; 4 Department of Molecular Biology, Medical Biochemistry and Pathology, Laval University, Quebec, Canada; 5 Université de médecine de Lille, Lille, France

**Keywords:** Lung cancer, Biomarkers, Oncology

## Abstract

With the increasing use of immune checkpoint inhibitors (ICIs), there is an urgent need to identify biomarkers to stratify responders and non-responders using programmed death-ligand (PD-L1) expression, and to predict patient-specific outcomes such as progression free survival (PFS). The current study is aimed to determine the feasibility of building imaging-based predictive biomarkers for PD-L1 and PFS through systematically evaluating a combination of several machine learning algorithms with different feature selection methods. A retrospective, multicenter study of 385 advanced NSCLC patients amenable to ICIs was undertaken in two academic centers. Radiomic features extracted from pretreatment CT scans were used to build predictive models for PD-L1 and PFS (short-term vs. long-term survivors). We first employed the LASSO methodology followed by five feature selection methods and seven machine learning approaches to build the predictors. From our analyses, we found several combinations of feature selection methods and machine learning algorithms to achieve a similar performance. Logistic regression with ReliefF feature selection (AUC = 0.64, 0.59 in discovery and validation cohorts) and SVM with Anova F-test feature selection (AUC = 0.64, 0.63 in discovery and validation datasets) were the best-performing models to predict PD-L1 and PFS. This study elucidates the application of suitable feature selection approaches and machine learning algorithms to predict clinical endpoints using radiomics features. Through this study, we identified a subset of algorithms that should be considered in future investigations for building robust and clinically relevant predictive models.

## Introduction

Lung cancer is one of the most aggressive cancer types with a dismal survival rate. Malignant epithelial tumors are broadly classified into non-small cell lung carcinoma (NSCLC) and neuroendocrine neoplasms. NSCLC accounts for 80% of primary pulmonary carcinomas, which are further divided into adenocarcinoma and squamous cell carcinoma. The five-year survival rate of lung cancer patients is just 21% for patients treated with pre-immunotherapy, largely due to the fact that most newly diagnosed patients (70%) present late in the evolution of the disease, when surgical treatment is impossible. In these patients, systemic treatments remained the only therapeutic option that were associated with significant toxicities and a poor response rate.

In recent years, immune checkpoint inhibitors (ICIs) have significantly modified lung cancer treatment options by providing new therapeutic avenues with superior efficacy and improved tolerability over traditional therapies^1,2^. PD-L1 is the only approved biomarker to guide a patient's treatment with ICIs, and currently impacts the clinical management of cancer based on the line of treatment, stage of the disease and the type of ICIs molecule used. For instance, patients with PD-L1-high tumors (expression value greater than 50%) are conventionally offered first-line monotherapy with anti-PD-1 antibodies, such as pembrolizumab. Although some patients with rapidly progressing disease may be treated with a chemotherapeutic regimen as a first line. In patients with low expression of PD-L1 (less than 49%), it is recommended that the patients are treated with a combination of platinum-based chemotherapy with pembrolizumab or atezolizumab as a first line^3,4^.

While the utility of PD-L1 biomarker was shown to be useful in both mono and combination immunotherapies, it has been found to have limitations in a subset of patients. In this context, there have been several efforts to develop OMICS- and imaging-based predictive biomarkers that could be used in addition or as a surrogate to PD-L1 staining to better identify the subgroup of patients most likely to benefit from these ICIs, thereby increasing progression-free survival (PFS) and overall survival (OS) of NSCLC patients^3–5^. Moreover, individualized treatments based on precise stratification to ICIs can improve the management of NSCLC patients. This has led to the search for predictive and prognostic biomarkers derived from clinical and imaging data^6^. In this regard, the availability of medical images has opened new frontiers for the application of radiomics and artificial intelligence (AI) techniques to build non-invasive biomarkers.

*Radiomics* aims to provide a detailed characterization that can quantitatively describe tumor features by leveraging medical imaging data such as CT and MRI scans. These features are extracted from a region of interest (ROI) annotated by the radiologist to build clinically relevant predictive models for various patient-specific outcomes. Several studies in the literature have focused on building imaging-based biomarkers for different tumor types. Along these lines, Zerunian et al.^7^ developed a radiomics signature using CT scans to predict OS and PFS in 21 patients with advanced NSCLC who were treated with first-line pembrolizumab. Trebeschi et al.^8^ built an imaging-biomarker to identify responders based on a lesion-based approach reflecting the metastatic condition of patients receiving ICIs. Sun et al. used baseline CT imaging data to develop a radiomics-based biomarker of tumor infiltration with CD8 cells in patients who received anti-PD-1 or anti-PD-L1 monotherapy^9^. In another study, Ligero et al. developed and validated a radiomics signature from pre-treatment CT scans to predict response to anti–PD-1/PD-L1 in patients with advanced solid tumors^10^. They integrated CT-based radiomics and clinical data into a multidimensional signature to predict the response. Recently, the multimodal predictive model integrating medical imaging, histopathologic and genomic features to predict PD-L1 was proposed by Vanguri et al.^11^. They developed the multimodal biomarker on a dataset of 247 patients with advanced NSCLC who received PD-(L)1-blockade-based therapy. Through extracting radiomics textural patterns from within and around the target nodules from the baseline CT images of advanced NSCLC patients undergoing anti-PDL1/PD1 monotherapy, Vaidya et al*.*^12^ built a predictive model to identify patients at high-risk for hyper progression. While only a subset of advanced NSCLC patients achieves a durable clinical response, the characteristics and predictors of long-term survivors to ICIs are not well known and may differ from short-term survivors, which is a pertinent clinical question that has not been addressed in the literature. This could help us to identify the patients with very short survival using non-invasive methods, thereby, making them amenable to other therapeutic strategies such as dose escalation or treatment combinations^13^. While the studies in the literature highlight the potential role for radiomics as a promising imaging-based biomarker in the management of NSCLC receiving ICIs^14,15^, however, there is no evidence yet of successfully building clinically translatable immunotherapy biomarkers. Moreover, none of these studies have attempted to systematically employ and compare a wide range of feature selection and machine learning methodologies for the prediction of PD-L1 and PFS (short-term vs. long-term survivors) in patients with advanced NSCLC treated with immunotherapy from a multicenter perspective using a large cohort of patients.

In this study, we investigate and perform a systematic comparison of a combination of feature selection strategies and machine learning approaches to develop imaging-based predictive biomarkers for PD-L1 and PFS (short-term vs. long-term survivors). Furthermore, in the present work, we used two independent cohorts from different institutions to develop and validate the radiomics biomarkers, which will have greater generalizability and translational ability. This would enable us to identify suitable feature selection and machine learning methods that can be used to develop clinically relevant, robust radiomics-based models for future studies.

## Materials and methods

### Description of cohorts

The study was performed on multicenter cohorts from two thoracic oncology reference centers, the Institut Universitaire de Cardiologie et de Pneumologie de Québec (Quebec Heart and Lung Institute, IUCPQ), and the Centre Hospitalier Universitaire de Montréal (CHUM), with the approval of their respective institutional ethics committees (MP-10-2020-3397/CÉR CHUM: 19.390). Ethical approval for the multicentre retrospective analyses of imaging and clinical data was obtained from the ethics committee of the two institutions, Comité d’éthique de la recherche de l’Institut Universitaire de Cardiologie et de Pneumologie de Québec and Comité d’éthique de la recherche du Centre Hospitalier Universitaire de Montréal. The inclusion criteria were selecting all those patients who have been diagnosed with advanced stage non-small-cell lung carcinoma and have received PD1 inhibitor immunotherapy treatment. And the exclusion criteria were removing the samples for whom there was an absence of informed consent for study and the unavailability of CT scans before and after immunotherapy. In this study, all methods were performed in accordance with the relevant guidelines and regulations. We conducted a retrospective study to evaluate PD-L1 expression and progression-free survival among patients diagnosed with NSCLC and treated with immune checkpoint inhibitors. The progression was defined by the qualitative evaluation of the radiologist. The samples were part of the Quebec Respiratory Health Network Tissue Bank (https://rsr-qc.ca/biobanque/) at the IUCPQ. Informed consent was obtained from all the study participants**.** In these cohorts, patients have been histologically diagnosed with an advanced stage of NSCLC and treated with PD-1/PD-L1 inhibitors. At least one breath-hold chest pre-treatment CT scan (3 months before the administration of ICIs) and one post-treatment CT scan were available to assess disease progression following the initiation of the treatment. The CHUM cohort (n = 223) was used for training purpose, while the IUCPQ cohort (n = 162) was used as the validation cohort. Out of the 385 patients, 326 of them have PD-L1 status. To develop the predictive models for PD-L1, the dataset included a total of 326 patients of which 192, 134 of them are from CHUM and IUCPQ cohorts, respectively. And, to build the predictive models for PFS, the dataset included 223 patients in the CHUM and 162 patients in the IUCPQ cohorts. Continuous and categorical data were compared by the Mann–Whitney U test and Chi-square (or Fisher’s exact) statistics, respectively.

### PD-L1 assessment

PD-1 is expressed on the surface of activated T-cells and down regulates T-cell activity upon binding to its ligands PD-L1 and PD-L2^16,17^. PD-L1 positive tumor cells (or tumor proportion score (TPS)) was evaluated by IHC staining (Dako Autostainer) using the 22C3 clone (pharmDx kit) as part of the routine patient management after lung cancer diagnosis. PD-L1 expression was assessed using the TPS, given as the percentage of tumor cells with positive membranous staining from 0 to 100%. Each tumor was reclassified according to the clinical cut-off for PD-L1 TPS, namely, < 1%, 1–49% and ≥ 50%. In the current work, we combined two categories, namely, < 1%, 1–49%, into a single group.

### PFS assessment

Progression-free survival (PFS) was defined as the time between the start of treatment and when the disease was progressed^18,19^. It is increasingly used as an important and even a primary endpoint in randomized cancer clinical trials in the evaluation of patients with solid tumors because of both practical and clinical considerations^20^. PFS is defined as the number of days/months from the first day of treatment to the date of disease progression, death for any cause, or the last follow-up (censored). Disease progression was confirmed imaging methods such as CT, MRI, or PET-CT^21^. In this study, we focused on building a classifier to predict short- and long-term progression-free survivors to ICIs. To carry out the model building for this task, we split both the CHUM and IUCPQ datasets into three quantiles. For the CHUM cohort, the first, second and third quantiles were 0.13–3.55, 3.55–11.5 and 11.5–67.3 months, respectively. For the IUCPQ dataset, the first, second and third quantiles were 0.13–2.27, 2.27–16.1 and 16.1–57.7 months, respectively. The first and last quantiles were utilized as PFS short-term and long-term survivors, respectively. We therefore considered this as a binary classification task. We excluded the patients with mid-term survival for the analyses.

### CT scan annotation

On de-identified 3-months pre-immunotherapy CT scans, each primary lesion was manually annotated by a radio-oncologist or radiologist. To determine the region of interest (ROI) for extraction of radiomic features, we followed a three-stage process: (i) After CT scan alignment, chest isolation was achieved through mathematical morphology-based denoising, and finally chest segmentation was performed using the connected regions; (ii) Following the detection of skin boundaries, the lung contour was roughly segmented and the pulmonary parenchyma was refined; and (iii) Based on the relative symmetry of the lung, the nodule ROI was identified that was independent of the size, position, and spreading near or through the pleura.

### Extraction of radiomics features

We used Pyradiomics (v 3.0.1), an open-source Python package for the extraction of Radiomics features from the ROI of pre-treatment CT scan data. ROI was made for accurate segmentation by a radiologist at IUCPQ and CHUM centers. These are interpretable, handcrafted features that are compliant with the Image Biomarker Standardization Initiative (IBSI). For normalization, slice thicknesses of all scans were interpolated to voxel sizes of 1 × 1 × 1 mm^3^ and features were calculated in 3D. The extracted features were grouped into four main categories, (i) intensity-based: which quantifies the nodules intensity characteristics using first-order statistics, calculated from the histogram of all voxel intensity values; (ii) shape-based: consists of features based on the shape of the lung nodules (such as sphericity or compactness); (iii) texture-based: features that can quantify the differences in the texture observable within the volume. These features were calculated in all three-dimensional directions within the tumor volume, thereby taking the spatial location of each voxel compared with the surrounding voxels into account; (iv) wavelet-based: calculates the intensity and textural features from wavelet decompositions of the original image, thereby focusing the features on different frequency ranges within the tumor volume^22,23^. These features were then used as input for building the predictive models.

### Feature selection methods

Radiomics studies typically contain hundreds of features^24^. The feature selection approaches are required to eliminate collinearity, reduce dimensionality, minimize noise, and avoid overfitting problems. Therefore, an initial data processing strategy is designed to limit the enormous number of features to a subset of the most relevant features. In this study, we applied a two-step feature selection methodology to develop the predictive models for PD-L1 and PFS. As a first step, we employed the least absolute shrinkage selection operator (LASSO) (Logistic regression with l1 penalty, and grid-search method was applied with a fivefold cross validation to obtain the hyperparameters) to reduce the dimensionality of the feature space^25^. Following that, five feature selection methods were employed to build the radiomics-based classifier, namely, analysis of variance (ANOVA)-F-test^26^ (AFT), Mutual Information^27,28^ (MI), ReliefF^29^ (RL), Surf^30^ (SF), and Multisurf^31^ (MSF), which are described below.

(i) Analysis of variance Anova F-test (AFT): it is a statistical test used to study the differences between both numerical and categorical sets of features of the data. The resulting F-statistic measure was used to rank each of the features in the dataset, and the features with the highest scores were selected as the optimal set of features^32,33^.
(ii) Mutual Information (MI): it is a method that is used to investigate the measure of dependence or mutual dependence between two given random variables. If the MI measure is close to 0, it indicates a weak association between the feature and the target^28,34^. If the input and the target are completely unconnected to one another, then we can say that they are independent of one another and that the input does not provide any information about the target. In this particular scenario, the amount of information shared between them is zero.
(iii) ReliefF (RL): this method is an extension of the original Relief-based algorithm^35^. In the original implementation of the Relief algorithm, the authors first selected a random instance from the data and then determined where that instance's nearest neighbor is, both in terms of the same class and the opposite class. A comparison is made between the values of the attributes of the sampled instance and those of the attributes of the nearest neighbors in order to update the relevance scores for each attribute. The idea behind this is that a useful characteristic should be able to discern between instances belonging to different classes while maintaining the same value for examples belonging to the same class^36^. Both RL and its modifications were implemented by Urbanowicz et al.^29^ as an open-source Python software package called scikit-rebate^37^. The package introduced a new core RBA called Multisurf (MSF) and Surf (SF) and extended all implemented algorithms to be able to handle different types of data problems, i.e. binary classification, multi-class classification, regression, discrete, continuous, or mixed feature types.

These feature selection algorithms do not explicitly identify the most significant features; instead, they produce a score for each individual feature. The top significant features were then selected based on the measured estimates obtained for each feature from each of the above algorithms, which was then used to build the classifier.

### Model development

**Figure 1:**
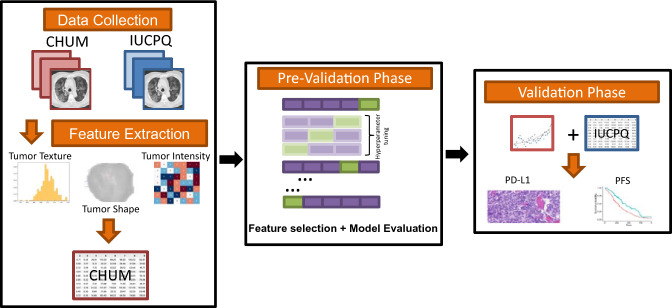
The workflow of radiomics analysis used in this study.

In our study, both PD-L1 and PFS were considered as binary classification tasks. For both tasks, we utilized seven different machine learning models: Adaptive boosting (AdaBoost), Decision Tree (DT), Random Forest (RF), Logistic Regression (LR), Support Vector Machines (SVM), K-nearest neighborhood (KNN), eXtreme Gradient Boosting (XGBoost). All classifiers were implemented using the Sklearn package, which makes it easy to use many machine-learning algorithms in Python. The analysis pipeline is illustrated in Fig. 1.

In general, we performed the following steps to develop the predictive radiomic-based models:

- Before building the models for PD-L1 and PFS, we removed all the samples that had any missing values. For PD-L1, we ended up with 192 and 134 samples in the CHUM and IUCPQ cohorts, respectively. For the PFS endpoint, there were no empty values.
- Data standardization: all variables were standardized with mean of zero and a standard deviation of one was done for both the cohorts.
- As a first step, we used Lasso Logistic regression with penalty ‘l1’ feature selection method. The Lasso is implemented on the whole training cohort independently of the other feature selections (AFT, MI, RL, SF, and MSF) and ML methods. We applied grid-search with fivefold cross validation to obtain the hyper parameters (i.e. to find the best C for Logistic regression). We then removed all features with coefficients equal to zero after training Lasso on the whole CHUM cohort. We ended up with 46 and 30 features after employing LASSO.
- Subsequently, each of the feature selection methods was combined with each of the seven machine learning classifiers, and, iteratively, the features were incremented to find a set of features that will result in the best model performance (in terms of Area under the curve (AUC)) for PD-L1 and PFS. We incrementally selected features (with those that were retained after the LASSO) for each of the feature selection methods.
- We first assessed the model performance in the pre-validation phase (i.e. on the discovery dataset), which consisted of a fivefold cross-validation with 10 repetitions for each of the models presented above. For each feature selection method, the best feature number was defined, which gave the highest score for PD-L1 and PFS. The classifiers hyperparameter tuning was also performed through cross-validation by using the GridSearchCV class of SciKit Learn. The feature selection and classification methods were implemented by using the SciKit Learn^38^ package in Python (scikit-learn version 1.0.2, Python version 3.9.13). Finally, we trained each of the models on the CHUM dataset (with the best features), validated them on the IUCPQ dataset, and computed the AUC score for PFS and PD-L1.

## Results

### Patient characteristics

The clinical data of all patients in the discovery and validation cohorts are summarized in Table 1. Continuous data were presented as mean ± standard deviation (SD), while categorical data were presented as counts and percentages.

**Table 1:** Clinical characteristics of discovery and validation cohorts.

Characteristics	PD-L1		PFS	
	CHUM (Discovery)	IUCPQ (Validation)	CHUM (Discovery)	IUCPQ (Validation)
Gender, n (%)				
Female	97 (50.5)	69 (51.4)	78 (52.3)	51(46.8)
Male	95 (49.4)	65 (48.5)	71 (47.6)	58(53.2)
Age (mean)	66.6 ± 8.9	67.8 ± 6.9	66.2 ± 8.73	67.9 ± 7.38
Smoking status, n (%)				
Former	126 (66.7)	89 (66.4)	93 (63.4)	75 (68.8)
Current	55 (29.1)	37 (27.6)	43 (29.5)	27 (24.8)
Never	8 (4.2)	8 (6.0)	10 (7.1)	7 (6.4)
ECOG status, n (%)				
0	67 (34.9)	36 (64.1)	53 (35.6)	29 (26.5)
1	93 (48.4)	82 (28.0)	69 (46.3)	66 (60.5)
2	28 (14.6)	7 (5.5)	23 (15.4)	6 (5.5)
3	4 (2.1)	3 (2.4)	4 (2.7)	2 (2.0)
				NA 6 (5.5)
PD-L1 expression, n (%)				
< 50%	95(50.1)	81 (60.5)		
> 50%	97 (49.9)	53 (39.5)	–	–
PFS, n, (%)				
Short-term			74 (50)	54 (50)
Long-term	–	–	75 (50)	55 (50)

We found no significant differences between the training and validation cohorts for both tasks (*p* value for PD-L1 and PFS was 0.11 and 0.34, respectively). Also, the range of progression-free survival was 0.13–67.3 months in the CHUM dataset and 0.13–57.7 months in the IUCPQ dataset. For the discovery cohort, the number of months for short-term survival and long-term survival was in the range of 0.13–3.55 and 11.5–67.3 months, respectively. And for the validation cohort, the range was 0.13–2.27 and 16.1–57.7 for the short- and long-term survival groups.

### Radiomics features

Using the Pyradiomics pipeline, a total of 851 radiomics features were extracted from the segmented tumor areas of the pre-treatment CT scans of two different cohorts in order to build predictive radiomics biomarkers. The CHUM cohort was utilized for feature selection and classification training, whereas the IUCPQ cohort was employed for evaluating the prediction performance for PD-L1 and PFS tasks.

### Predictive performance of the feature selection and classification methods

**Figure 2:**
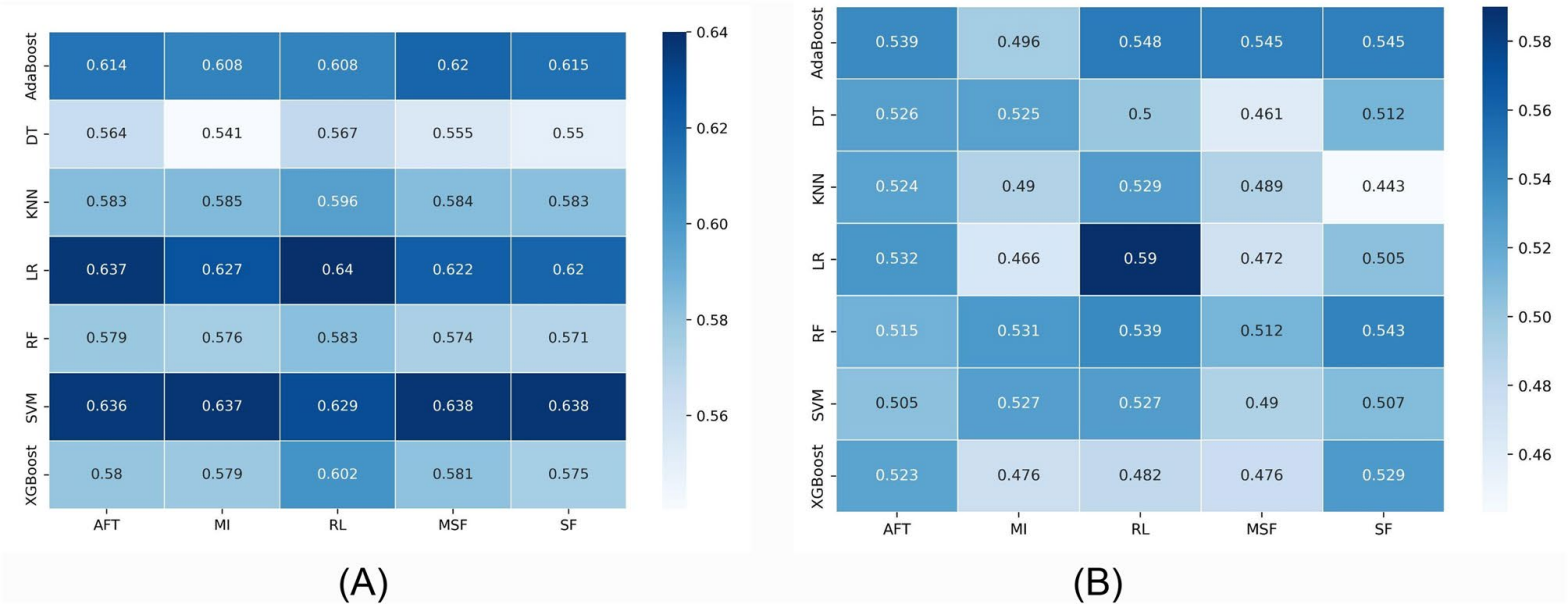
Heatmaps illustrating the performance of each machine learning algorithm (rows) with each feature selection method (columns) for the PD-L1 task. (**A**) AUC scores in the cross-validation phase and (**B**) AUC scores in the validation phase.

**Figure 3:**
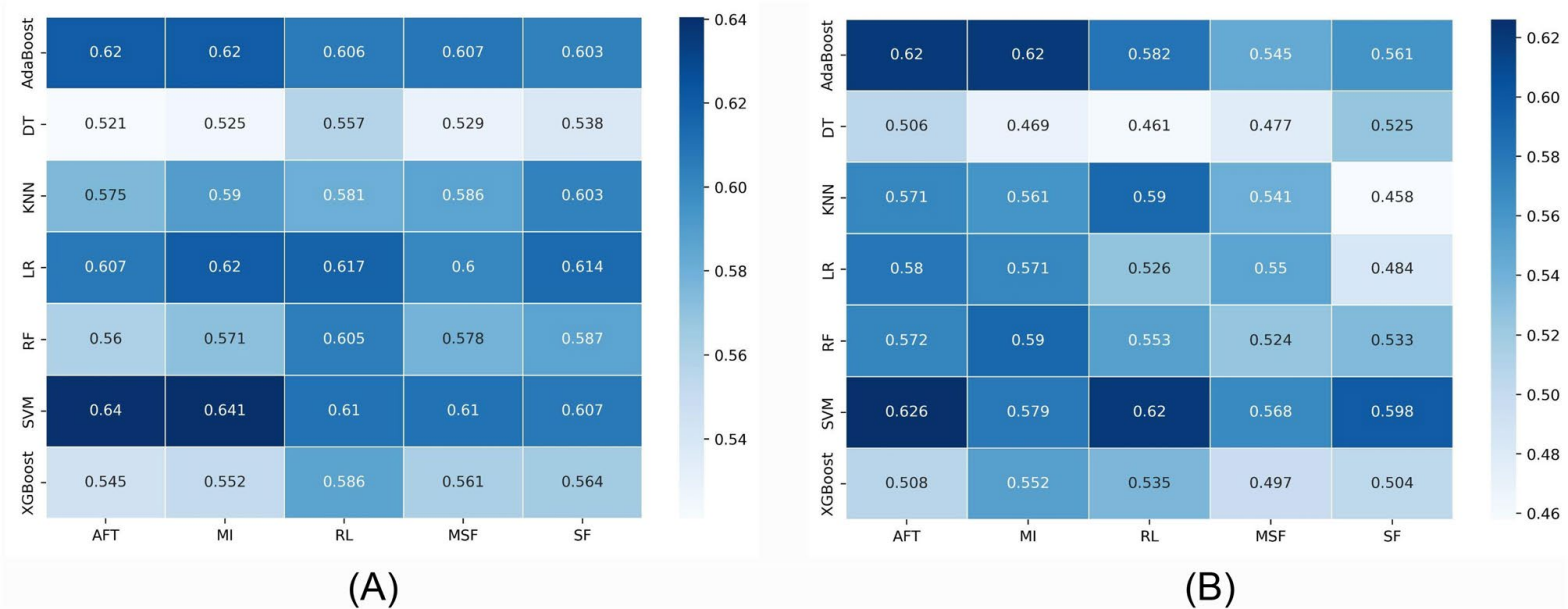
Heatmaps illustrating the performance of each machine learning algorithm (rows) with each feature selection method (columns) for the PFS task. (**A**) AUC scores in the cross-validation phase and (**B**) AUC scores in the validation phase.

The predictive performance of different feature selection and classification methods was assessed using AUC. Figures 2 and 3 depict the performance of feature selection methods (in columns) and machine learning classifiers (in rows) for PD-L1 and PFS, respectively. For each classification method, there were five AUC values corresponding to the five different feature selection methods. Figure 2A and B present AUC scores for the discovery and validation cohorts, respectively, for PD-L1. Two models, LR and SVM, showed similar performance on the discovery cohort (AUC = 0.62–0.64); however, the RL feature selection method with LR showed the best performance on the validation cohort (AUC = 0.59). The worst performance was shown by the KNN model with the SF feature selection method on the validation cohort (AUC = 0.443). In general, we found that the XGBoost and KNN performed poorly with a few feature selection methods where the AUC scores were less than random.

Figure 3A and B present the AUC for PFS on the discovery and validation cohorts, respectively. The performance of the LR, AdaBoost, and SVM models with all feature selection models was found to be better than the others in the discovery cohort, with the AUC score higher than 0.60. SVM with the AFT feature selection method resulted in the highest AUC on validation cohort (AUC = 0.626). The worst performance was shown by the KNN model with the SF feature selection method on the validation dataset with AUC = 0.458. In general, AdaBoost and SVM classifiers showed better performance than others on the test dataset.

### Median performance of feature selection and machine learning methods

**Figure 4:**
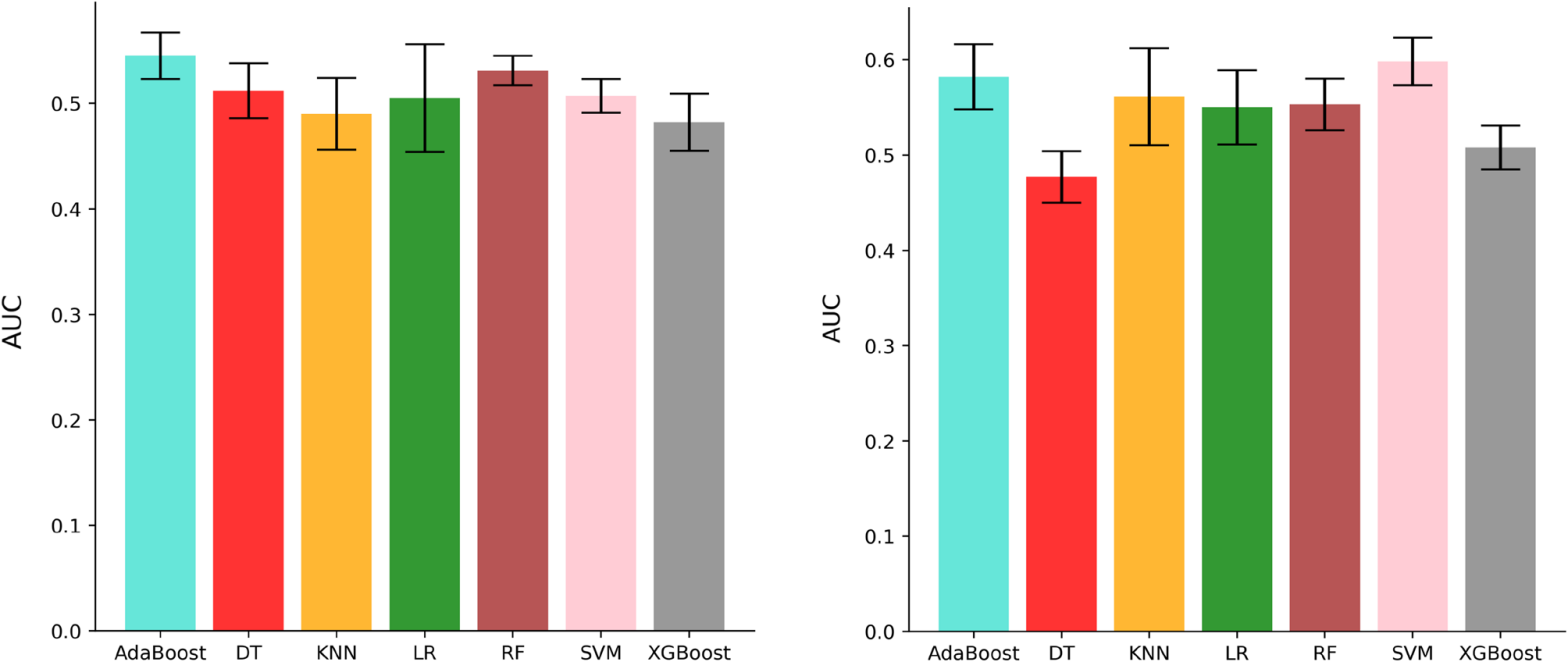
Median performance of machine learning methods to predict PD-L1 and PFS (short-term vs. long-term survivors) on the validation dataset.

We also computed the median performance of the classifiers across all feature selection methods for PD-L1 and PFS, which is displayed in Fig. 4. For PD-L1, the highest median performances were obtained by the AdaBoost (AUC: 0.545 ± 0.022, median ± std), followed by the RF (AUC: 0.531 ± 0.014, median ± std). For PFS, the highest median performances were obtained by SVM (AUC: 0.598 ± 0.025), followed by AdaBoost (AUC: 0.582 ± 0.034).

The lowest model performance was achieved by DT to predict both PD-L1 and PFS. Most importantly, we found several combinations of feature selection methods and machine learning algorithms to achieve a similar median score for both tasks.

## Discussion

Treatments for patients with advanced NSCLC are non-curative and thus intended to prolong their survival while minimizing treatment-induced toxicities^39^. In the past few years, treatments harnessing the immune system have drastically modified the therapeutic management of NSCLC, notably with the use of ICIs. These compounds facilitate the recognition of tumor cells by the immune system, enabling immune cells to eliminate them more effectively. Despite their promising success, clinical benefit remains limited to only a subset of patients. To date, PD-L1 is the only approved conventional biomarker used to guide a patient's treatment with ICIs. Albeit, this is based on several clinical factors such as histology, first line or second line of treatment, the stage of the disease and the type of immunotherapy compound used. While its utility was shown in both mono and combination immunotherapies, PD-L1 does not have the full ability to stratify patients who would benefit from immunotherapy^40^. In this context, there have been several efforts to develop OMICS-based biomarkers using gene expression data, however, none of these predictive gene expression-based signatures have been translated to clinical use^10,41^. Translation of OMICS-based biomarkers to clinical care requires a rigorous model development and validation process. The Institute of Medicine provided recommendations concerning the clinical utility to regulatory issues on OMICS-based methods^42,43^.

While data-driven OMICS-based biomarkers are now being evaluated in immunotherapy^41^ and in other therapeutic interventions^44^, there continues to be a need to identify noninvasive biomarkers to predict and monitor response to ICIs. This has spurred research towards building imaging-based prognostic and predictive biomarkers leveraging medical imaging data. A few studies have developed imaging-based biomarkers in NSCLC patients treated with ICIs, mostly in the context of predicting response to ICIs in a metastatic setting, or overall survival and assessing CD8 cells in tumor. To better stratify the subgroup of patients who will most likely benefit from these ICIs, radiomic biomarkers are being developed so that they can be used as a surrogate to PD-L1 expression as well as to predict PFS and OS of NSCLC patients. However, none of them have been successfully translated to clinical practice. Also, the predictive power of these models has not been validated in multi center settings. Nevertheless, it is not clear whether any of the feature selection methodologies combined with learning algorithms in the literature have led to models with the highest predictive accuracy.

Although prior immunotherapy studies in the literature have highlighted the role of data-driven biomarkers leveraging medical imaging data in the management of NSCLC patients^7–9,12,13^, they have several limitations. One limitation is the incorporation of radiomic information in the form of univariate feature predictors. Importantly, most of these studies have not validated the predictive power of these radiomic models in the context of multicenter cohorts. Furthermore, in radiomics, feature selection is used to reduce the dimensionality of the feature space, which is subsequently used to build prognostic or predictive signatures using machine learning algorithms. Although a variety of feature selection and machine learning approaches exist, most of the radiomics-based studies considered the combination of a single feature selection with one learning algorithm^8^. And, to date, it is not clear whether any of the methodologies in these studies have led to models with the highest predictive accuracy. Furthermore, none of these studies have attempted to do a systematic comparison of a wide range of feature selection and machine learning methodologies to predict PD-L1 and PFS in a multi-institutional setting on larger cohorts across hospital systems for the purpose of translatability and clinical applicability.

Based on this premise, in the present study, we attempted to explore the application of several feature selection approaches and machine learning algorithms to predict PD-L1 and PFS by leveraging radiomics features from CT-scan data. For this purpose, we used five feature selection methods, namely, AFT, MI, RL, SF, and MSF, and seven machine learning algorithms: AdaBoost, DT, RF, LR, SVM, KNN, and XGBoost. Both PD-L1 and PFS were defined as a binary classification in this study. In the PD-L1, the performance of LR, SVM, and AdaBoost was found to be almost similar in the discovery cohort. For the validation cohort, the RL feature selection approach with LR was found to have the highest performance (AUC = 0.59) among all combinations of other machine learning models and feature selection methods. We observed that the XGBoost and KNN algorithms fared poorly with the majority of the feature selection strategies. Furthermore, AdaBoost, SVM, and LG demonstrated better performance to predict the PFS on the discovery cohort. For the validation cohort, AdaBoost with the AFT and MI and SVM with the AFT and RL received the best AUC score (AUC = 0.62–0.626). The strengths of our study include the large number of patients evaluated on the two institutional cohorts (n = 385) considering the heterogeneity of data across the two hospital centers as well as the use of two clinical endpoints PD-L1 and PFS (short-terms vs. long-term survivors).

Nevertheless, we do acknowledge the limitations of our study that include the following: current study was limited to primary tumors only, we did not fully account for all potential causes of bias in the study design, assessing the PD-L1 status at variable time points in regard to immunotherapy date of treatment, heterogeneity of scanners across hospitals along with the impact of gray level discretization on the radiomics feature extraction. As the scanning techniques are constantly evolving, it is possible to have a shift in the radiomics feature distribution that occurs as a result of these variations. These limitations can, in part, be addressed by the incorporation of harmonization approaches to combat scanner variations (such as the acquisition parameters) as well as considering robust and reproducible radiomics features extracted from multiple IBSI compliant radiomics pipelines^45^. Furthermore, the classifier for the PFS was built only for the extreme survival groups of patients. We hypothesize that by addressing the above limitations, the developed imaging-based models can be further improved to predict the clinical endpoints, PD-L1 and PFS (short-term vs. long-term survivors), which is a subject of ongoing investigation.

In conclusion, a wide range of feature selection methods and machine learning approaches are useful in radiomics studies to increase the robustness of imaging-based predictive models. Most importantly, it is crucial to standardize the radiomics processing methods or to select only reproducible features across radiomics platforms that could potentially aid in building stable predictive models to ICIs.

## Data Availability

Data presented in this study are not publicly available at this time but may be obtained from the corresponding author, Venkata Manem upon reasonable request.
